# The *MUC5B* Variant Is Associated with Idiopathic Pulmonary Fibrosis but Not with Systemic Sclerosis Interstitial Lung Disease in the European Caucasian Population

**DOI:** 10.1371/journal.pone.0070621

**Published:** 2013-08-05

**Authors:** Raphael Borie, Bruno Crestani, Philippe Dieude, Hilario Nunes, Yannick Allanore, Caroline Kannengiesser, Paolo Airo, Marco Matucci-Cerinic, Benoit Wallaert, Dominique Israel-Biet, Jacques Cadranel, Vincent Cottin, Steven Gazal, Anna L. Peljto, John Varga, David A. Schwartz, Dominique Valeyre, Bernard Grandchamp

**Affiliations:** 1 Service de Pneumologie A, APHP, Hopital Bichat, Paris, France; 2 INSERM, Unité 700, Faculté Bichat, Université Paris 7, Paris, France; 3 Université Paris 7 Denis Diderot, Paris, France; 4 Service de Rhumatologie, APHP, Hopital Bichat, Paris, France; 5 Service de Pneumologie, APHP, Hopital Avicenne, Bobigny, France; 6 Service de Rhumatologie, APHP, Hopital Cochin, Paris, France; 7 Service de Génétique, APHP, Hopital Bichat, Paris, France; 8 Rheumatology and Clinical Immunology, Spedali Civili, Brescia, Italy; 9 Department of Biomedicine, Section of Rheumatology, University of Florence, Florence, Italy; 10 Service de Pneumologie, CHRU de Lille, Lille, France; 11 Service de Pneumologie, Hopital Européen Georges Pompidou, Paris, France; 12 Service de Pneumologie, Hopital Tenon, Paris, France; 13 Service de Pneumologie, Hopital Louis Pradel, Lyon, France; 14 Université Charles de Gaulle – Lille III, Groupe de Recherches 'Modélisation Appliquée à la Recherche en Sciences Sociales (GREMARS), Lille, France; 15 Departments of Epidemiology, School of Public Health, University of Colorado, Denver, Colorado, United States of America; 16 Northwestern University Feinberg School of Medicine, Chicago, Illinois, United States of America; 17 Department of Medicine, School of medicine, University of Colorado, Denver, Colorado, United States of America; University Medical Center Freiburg, Germany

## Abstract

A polymorphism on the *MUC5B* promoter (rs35705950) has been associated with idiopathic pulmonary fibrosis (IPF) but not with systemic sclerosis (SSc) with interstitial lung disease (ILD). We genotyped the *MUC5B* promoter in the first 142 patients of the French national prospective cohort of IPF, in 981 French patients with SSc (346 ILD), 598 Italian patients with SSc (207 ILD), 1383 French controls and 494 Italian controls. A meta-analysis was performed including all American data available. The T risk allele was present in 41.9% of the IPF patients, 10.8% of the controls (P = 2×10^–44^), OR 6.3 [4.6–8.7] for heterozygous patients and OR 21.7 [10.4–45.3] for homozygous patients. Prevalence of the T allele was not modified according to age, gender, smoking in IPF patients. However, none of the black patients with IPF presented the T allele. The prevalence of the T risk allele was similar between French (10%) and Italian (12%) cohorts of SSc whatever the presence of an ILD (11.1% and 13.5%, respectively). Meta-analysis confirmed the similarity between French, Italian and American cohorts of IPF or SSc-ILD. This study confirms 1) an association between the T allele risk and IPF, 2) an absence of association with SSc-ILD, suggesting different pathophysiology.

## Introduction

Lung fibrosis is a common trait of idiopathic pulmonary fibrosis (IPF) and systemic sclerosis (SSc) with interstitial lung disease (ILD). Indeed, ILD is present in almost 40% of the patient with SSc and is the major cause of death during SSc [1]. However the most common histological and radiological pattern observed in SSc-ILD is non specific interstitial pneumonia (NSIP), whereas the pattern associated with IPF is usual interstitial pneumonia (UIP) [2]. There is still a debate concerning the pathophysiology of NSIP and UIP, there is evidence that both patterns share aetiologies and can occur in the same context.

Both IPF and SSc are considered as genetic complex diseases, and occur in genetically predisposed individuals who have experienced certain environmental or stochastic stimuli [3]. An association between a functional polymorphism located in the putative promoter region of the *MUC5B* gene (rs35705950) and sporadic and familial IPF has been recently identified in 3 American cohorts including 916 patients [4], [5] and was recently confirmed in 2 genome wide association studies [6], [7]. A candidate gene association study which investigated *MUC5B* rs35705950 in 109 individuals having SSc-ILD suggested a lack of association [8]. Moreover a recent gene association study in England confirmed an association with IPF (n = 110) and a lack of association with SSc-ILD (n = 440) or sarcoidosis (n = 180) [9]. To date, no information is available regarding the association between *MUC5B* rs35705950 and IPF or SSc-ILD in French or Italian populations. These findings prompted us to test an association between the *MUC5B* rs35705950 variant and i) IPF in the French population, and ii) SSc-related ILD in two large European Caucasian populations. We then performed a meta-analysis of the American and European data in IPF and SSc.

## Methods

### Study populations

#### IPF populations

A French prospective and multicentric cohort of recently diagnosed IPF (the COFI cohort) was established starting in 2008. Inclusion criteria comprised a diagnosis of IPF based upon either surgical biopsy or a characteristic CT scan pattern according to the 2001 ATS/ERS consensus [10]. The first imaging allowing the diagnosis of IPF had to date back to a maximum of 9 months prior to inclusion. All demographic, comorbidity, clinical and functional data were prospectively and serially recorded.

#### SSc populations and controls

We studied two European cohorts of patients with SSc and their associated controls. The French cohort included 981 SSc patients and 1229 controls coming from the French network as previously described [11]. The replication step came from Italy included 598 SSc patients and 494 controls [11]. Of note all SSc patients and controls were Caucasian. Patients were classified according to LeRoy's cutaneous subtypes [12]. SSc patients were tested for antinuclear antibodies (ANA) using indirect immunofluorescence (IIF) and HEp-2 cells as antigen substrate (Antibodies Inc., Davis, CA). Specific SSc antibodies were systematically assessed; anti-centromere antibodies (ACAs) were determined by their distinctive IIF pattern on HEp-2 cells. Anti-topoisomerase I antibodies (TOPO) were determined by counter immuno-electrophoresis.

ILD was defined as the presence of ground glass opacity and/or reticular opacities in a peripheral distribution on chest CT scan, however a classification according to the UIP or NSIP pattern was not performed. CT scan was not available for 99 and 7 patients of the French and Italian cohorts respectively.

Local institutional review board approval was obtained for every study subject and written informed consent was obtained from all subjects (Ethical Committee of Firenze, Comité pour la Protection des Personnes Ile de France X, Aulnay sous Bois).

### Genotyping

All the subjects were genotyped for the *MUC5B* rs35705950 SNP using a competitive allele specific PCR system (Kaspar genotyping, Kbioscience, Hoddeston, UK) and Taqman SNP genotyping assay-allelic discrimination method (Applied Biosystem, Foster City, CA) as previously described [11]. The average genotype completeness was 99% for SSc and IPF and controls samples for the SNP investigated. The accuracy was >99%, according to duplicate genotyping of 10% of all samples.

### Statistical analysis

The statistical analyses were performed using the R computer package software (version 2.10.1). The level of significance for all the tests corresponds to a type-I error-rate α = 5%. Tests for conformity to Hardy–Weinberg equilibrium (HWE) were performed using a standard χ 2 test (1 degree of freedom) to test for differences between observed and expected genotype distributions based on control population allele frequencies.

Individual association analyses of the *MUC5B* rs35705950 SNP with IPF or SSc were performed by comparing cases and controls with a Fisher's exact or Chi2 test on genotypes. Blacks individuals with IPF were excluded from analysis as our controls were all Caucasians. The corresponding ORs were assessed using a standard logistic regression analysis with the most frequent homozygous genotype in the control population taken as the reference. The same procedure was applied in subgroups stratified according to SSc phenotypes, compared to controls.

### Meta-analysis of rs35705950

We performed a systematic review and the meta-analysis of all data published up to December 2012. We searched Medline via PubMed with the terms “MUC5B” for articles published in English that provided 3 relevant articles. We did not find other articles with hand-searched reference lists of clinically relevant articles. We contacted the corresponding author that provided us the full data. We therefore fulfilled the PRISMA guidelines.

#### Idiopathic pulmonary fibrosis

The meta-analysis included the data obtained in the French IPF population from this study, and the data from American IPF patients coming from Denver (n = 488), Chicago (n = 95) and Pittsburgh (n = 246), previously published [4], [5].

#### Systemic sclerosis

The meta-analysis included data obtained in the French and Italian populations from this study, and data from American population of SSc patients from Northwestern Scleroderma Program (n = 231) and controls from Denver (n = 322), Chicago (n = 636) and Pittsburgh (n = 166) [4], [5], [8].

The combined data including the 4 populations of IPF, 3 populations of SSc and 5 populations of controls were analyzed by calculation of homogeneity of ORs among the cohorts using the Breslow-Day and Woolf Q methods, and by calculation of the pooled ORs under a fixed-effects model (Mantel-Haenszel metaanalysis) or random-effects model (DerSimonian-Laird) when necessary and assessed by logistic regression analysis genetic effects under 3 modes of inheritance: additive, dominant, and recessive.

## Results

### Idiopathic Pulmonary Fibrosis

The demographic and clinical characteristics of the subjects are summarized in Table 1. The genotypic frequencies for rs35705950 were consistent with in Hardy–Weinberg equilibrium in the controls populations.

**Table 1:** Characteristics of the French IPF patients.

	French Population N 142
Female	18%
Age, years	69.8±8.9
Smoker (active)	68% (4%)
Ethnic status	
Caucasian	
European	82%
North African	14%
Black	4%
Pulmonary Fonction	
FVC (%)	76±20
DLCO (%)	47±19

Data are expressed as % or mean± SD. IPF Idiopathic pulmonary fibrosis.

Case-control analysis shows association of the rs35705950 SNP with IPF (Table 2). The minor-allele frequency was 41.9% in the IPF cohort compared to 10.8% in controls (P = 2.9×10^−44^). Odds ratios for IPF in subjects who were heterozygous and homozygous for the minor allele of rs35705950 were 6.3 (95% confidence interval, [CI], 4.6 to 8.7) and 21.7 (95% CI, 10.4 to 45.3) respectively.

**Table 2:** Association between the *MUC5B* rs35705950 polymorphism and IPF, SSc and SSC-ILD in the European population.

	TT (%)	GT (%)	GG (%)	T (%)		*P*	OR [95% CI]
**French populations**							
**IPF** n = 142	11.90	53.57	34.52	38.69	GT TT	*4*×*10* ^−*29*^ *9*×*10* ^−*29*^	6.4[4.5–9]19[9–36]
**SSc** n = 981	1.22	17.53	81.24	9.99	G GG GT	*0.36* *0.61* *0.42*	0.91[0.76–1.11]0.83[0.40–1.71]0.92[0.74–1.14]
**SSc-ILD+** n = 346	1.39	19.50	79.11	11.14	G GG GT	*0.79* *0.95* *0.74*	1.04[0.79–1.34]0.97[0.36–2.61]1.05[0.78–1.41]
**SSc-ILD-** n = 536	0.93	17.72	81.34	9.79	G GG GT	*0.36* *0.63* *0.93*	0.89[0.71–1.13]0.63[0.24–1.70]0.93[0.72–1.20]
**Controls** n = 1383	1.45	18.73	79.83	10.81			
**Italian populations**							
**SSc** n = 598	1.34	21.40	77.26	12.04	G GG GT	*0.82* *0.54* *0.55*	1.03[0.79–1.34]0.75[0.29–1.95]1.09[0.81–1.47]
**SSc-ILD+** n = 207	1.45	24.15	74.40	13.53	G GG GT	*0.35* *0.79* *0.21*	1.17[0.84–1.65]0.84[0.22–3.14]1.28[0.87–1.82]
**SSc-ILD-**n = 384	1.30	19.79	78.91	9.79	G GGGT	*0.72* *0.54* *0.95*	0.9[0.71–1.13]0.71[0.24–1.70]0.99[0.72–1.20]
**Controls** n = 494	1.82	19.84	78.34	11.74			

**Figure 1:**
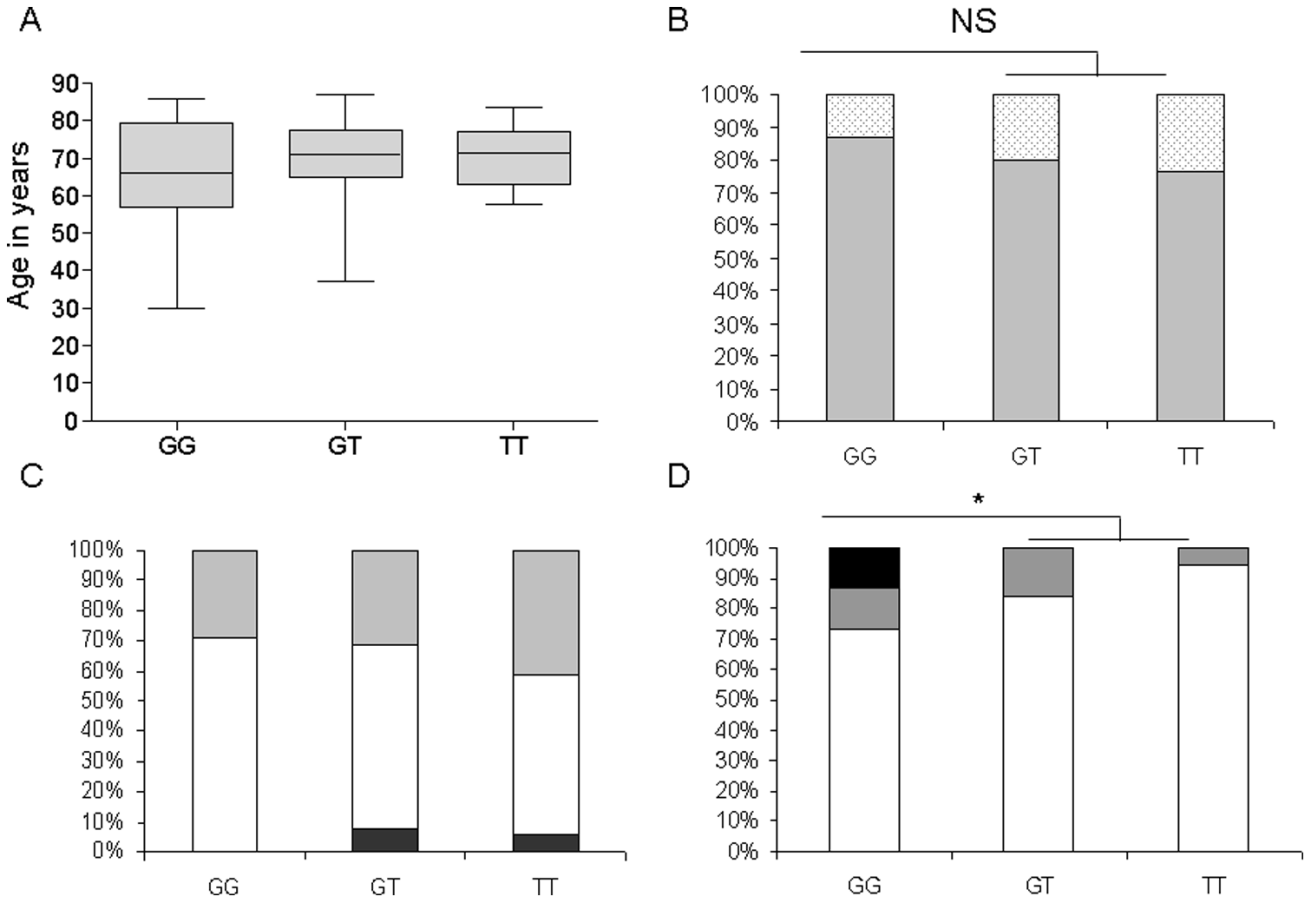
Distribution of clinical characteristic according to the *MUC5B* genotype. (A) Distribution of age according to genotype. The centre line of the box denotes the mean, the extremes of the box the interquartile range and the bars the highest and lowest values. (B) Distribution of the gender according to the genotype. Percentage of men is represented in grey; percentage of women is represented in white with black dots. NS non significant. (C) Distribution of the smoking status according to the genotype. Percentage of never smokers is represented in grey, percentage of past smokers is represented in white, and percentage of active smokers is represented in black. (D) Distribution of the ethnic status according to the genotype. Percentage of European is represented in white, percentage of North African is represented in grey, and percentage of black patients is represented in dark. * P = 0.0008.

In the IPF cohort, the distribution of homozygous and heterozygous patients was not different according to gender, age, smoking habitus, pulmonary function test or presence of a cancer at diagnosis, even in subgroup analysis according to gender (Figure 1). The MUC5B rs35705950 IPF risk allele was not detected in the French black individuals with IPF (P = 0.0008).

### Systemic sclerosis

The demographic and clinical characteristics of the subjects are summarized in Table 3.

**Table 3:** Characteristics of the SSc patients in the 2 investigated European populations.

	French Population n = 981	Italian Population n = 598
Female (%)	83.6	86.1
Mean age, years	57.2±12.8	53.6±10.9
Mean disease duration, years	10.9±8.7	11.5±8.9
Limited cutaneous subtype (%)	64.5	69.9
Anti-Topo I positive patients (%)	27.3	29.9
ACA positive patients (%)	40.3	42.1
ILD on CT scan (%)	35.2%	34.6%

Anti-Topo I: anti-topoisomerase I antibodies; ACA: anti-centromere antibodies; ILD interstitial lung disease. Data are expressed as % or mean ± SD. SSc systemic sclerosis. ILD Interstitial lung disease.

Regarding the French population of SSc, no allelic association was detected between the *MUC5B* rs35705950 SNP and the overall disease: the minor allele was found in 10% of SSc individuals compared to 10.8% in controls (P = 0.36). We failed to detect any statistical difference of the genotypes distribution between SSc patients with and without ILD and the controls in the French population (Table 2). Similar allelic and genotypic frequencies were observed in the Italian population, the lack of association between SSc with or without ILD and *MUC5B* rs35705950 being replicated (Table 2). In the SSc cohorts, the distribution of homozygous and heterozygous patients was not different according to gender.

### Meta-analysis

The demographic and clinical characteristics of the subjects are summarized in Table 4.

**Table 4:** Characteristics of the populations investigated for meta-analysis.

	IPF	Controls for IPF	SSc	SSc-ILD+	Controls for SSc
N	975	2353	1810	662	2045
Female (%)	26.8	55.5*	89.9	83.6	62.4*
Caucasian (%)	100	100	na	na	100
Mean age, years	68.7	48.0*	54.5	56.5	45.2*
FVC (%)	66.9*	na	85.5*	82.4*	NA
DLCO (%)	45.9*	na	65.1*	54.9*	NA

* some data may be missing but data expressed represent always more than 2/3 of the populations. na not available.

**Figure 2:**
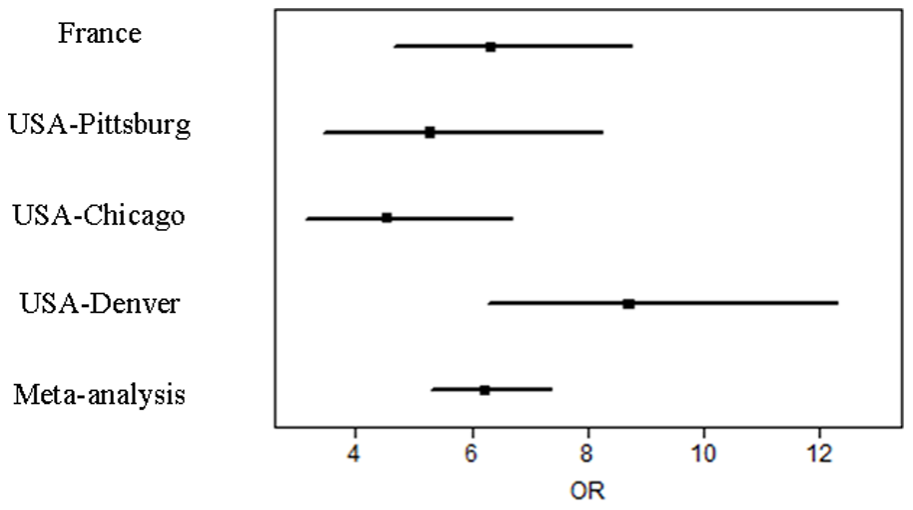
Meta-analysis of the *MUC5B* rs35705950 T allele risk in the three American cohorts and the French cohort of idiopathic pulmonary fibrosis (IPF). Forrest plots of the meta-analysis of the association of the *MUC5B* rs35705950 T polymorphism and IPF. Bars represent the 95% interval.

Remarkably, the genotypic frequencies were very similar in the French and American populations of IPF and controls. The meta-analysis of the 4 IPF populations evidenced a strong association between *MUC5B* rs35705950 T allele and IPF: P = 5×10^−105^, OR 6.2 95% CI[5.3–7.3] (Figure 2).

**Figure 3:**
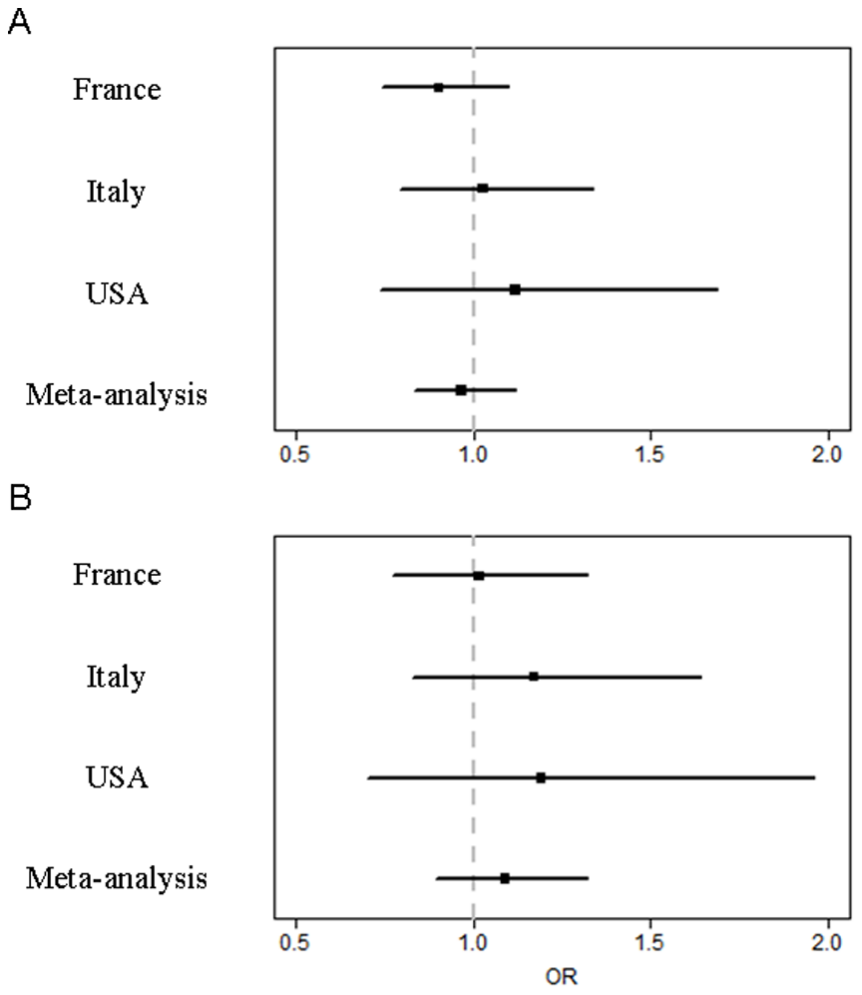
Meta-analysis of the *MUC5B* rs35705950 T allele risk in the two European (French and Italian) cohorts and the American cohort of systemic sclerosis (SSc). Forrest plots of the meta-analysis of the association of the *MUC5B* rs35705950 T polymorphism and (A) all SSc and (B) SSc with interstitial lung disease. Bars represent the 95% interval.

The genotypic frequencies of the herein study were very similar to that observed in the North American populations of SSc and SSc-ILD. The meta-analysis of the 3 SSc populations confirmed the absence of association between *MUC5B* rs35705950 T allele and SSc whatever the presence of an ILD, OR 0.97 (95% CI, 0.84 to 1.12), P = 0.64, for the association with SSc, and OR 1.09 (95% CI, 0.9 to 1.32), P = 0.38, for the association with SSc-ILD (Figure 3).

## Discussion

In this study, we confirm in European populations an association between the *MUC5B* rs35705950 T allele and IPF and a lack of association with SSc related ILD. Despite a relative low number of IPF patients included, the meta-analysis provides a definitive conclusion about this association in Caucasian population. Interestingly, we observed that this polymorphism was absent in the six black patients with IPF.

Our meta-analysis provides a more accurate evaluation of the IPF risk associated with the T allele in Caucasian population. Remarkably the prevalence of the T allele risk in the cohorts of controls, SSc, SSc-ILD or IPF was almost similar in every population, including the English cohorts [9]. Indeed these cohorts are large and probably representative of the entire Caucasian population.

The Odd Ratio for IPF in heterozygotous carriers of the T allele is 6, whereas almost 10% of the control population presents with the T allele. The exact role of this polymorphism in IPF pathophysiology remains to be determined. Seibold *et al.* suggested that rs35705950 was functional [4]. Indeed, in unaffected subjects, the presence of the T allele was associated with a 37 fold increased expression of *MUC5B* gene in the lung. Furthermore, *MUC5B* expression was increased 14-fold in the lung in IPF patients when compared to controls. MUC5B is the dominant gel-forming mucin in the normal distal airway epithelium. Plantier and colleagues demonstrated by immunohistochemistry that, in contrast to chronic obstructive pulmonary disease, *MUC5B* was the predominant mucin detected in the abnormal mucus cells observed in the honeycombing areas of the fibrotic lung in patients with IPF [13]. This result was confirmed by Seibold and coll. [14]. Interestingly a trend was observed by Stock and colleagues between the *MUC5B* variant and slower decline in forced vital capacity, whereas no difference was evidenced in this cohort regarding age or severity at diagnosis between carriers or non carriers of the T allele risk [9]. Some have suggested that a therapy targeting *MUC5B* transcriptional activity should be evaluated in IPF [15]. The association between increased expression of *MUC5B* and IPF suggest that MUC5B may have a direct role in the pathogenesis of IPF. MUC5B may interfere with the normal repair process of the alveolar epithelium. For instance, MUC5B overexpression leads to an aggressive behavior of breast cancer MCF7 cells with increased proliferation and invasion in vitro, although the mechanisms involved are unknown [16]. One may speculate whether MUC5B overexpression stimulates a fibroproliferative response, or associates with abnormal mucosal defences to exogenous injury. Very recently, the *MUC5B* rs35705950 T allele has been shown to be associated with better survival among patients with IPF [17]. Further studies are clearly needed to better understand this elective link between MUC5B overexpression and IPF and the absence of link with other fibrotic lung diseases.

Our meta-analysis clearly suggests that the T allele is not associated with an increase risk of SSc or ILD in SSc in the Caucasian population. This is a very important result as it may shed some light on specific lung fibrotic process in SSc. The radiological and pathological pattern in IPF is UIP. In SSc, different patterns have been described but the prominent pattern is NSIP [2], [18], [19]. To date, the SSc-ILD pathogenesis remains poorly understood. If the exact genetic contribution to SSc-ILD remains unknown, a population-based study provided evidence for the heritability of ILD in SSc, first-, third-, and fourth-degree relatives of individuals with SSc having significantly elevated relative risks for ILD [20]. In line with this, it has been previously identified that some of the SSc risk variants, such as *IRF5* rs20046640, *STAT4* rs7574865 and *NLRP1* rs8182352 susceptibility alleles, contribute to a disease-specific phenotype, notably SSc-ILD [11], [21], [22]. The lack of an association between the *MUC5B* rs35705950 T allele and SSc related ILD suggest that this polymorphism does not associate with lung fibrosis in general, but might be specific for either IPF or UIP. Evaluating the prevalence of *MUC5B* polymorphism in large cohorts of idiopathic or non-idiopathic ILD may improve our understanding of the pathophysiology of these diseases.

Our IPF population included only 6 black patients with IPF; the *MUC5B* polymorphism was absent in all of them. There is paucity of data regarding prevalence of IPF in black populations. In the 3 American cohorts of IPF evaluating the prevalence of *MUC5B* polymorphism, all subjects were white [4], [5]. Nathan and al. reported a 13.8% prevalence of black in their IPF cohort from Fairfax [23], whereas Swigris and al. recently reported twice less IPF and a decreased risk of death from IPF in black descendents [24]. Further studies including a re-sequencing of MUC5B in other populations than Caucasian are required to better evaluate the contribution of *MUC5B* in the genetic IPF background in distinct populations. Distribution of T allele risk was not different according to gender. However woman represent only 18% of the cohort, that is less than the 28% of the Fairfax cohort [23].

Altogether, European data and meta-analysis confirm a strong association between the *MUC5B* rs35705950 variant and IPF in Caucasian population whereas this association was absent in SSc-related ILD. Further studies are required to evaluate this genetic susceptibility marker as a prognosis factor in IPF.

## Supporting Information

Flowchart S1 **Prisma flowchart.**
(DOC)

Guidelines S1 **Prisma guidelines.**
(DOC)
