# Supplementary material for: The MUC5B Variant Is Associated with Idiopathic Pulmonary Fibrosis but Not with Systemic Sclerosis Interstitial Lung Disease in the European Caucasian Population
Source: PLoS One. 2013 Aug 5;8(8):e70621. doi: 10.1371/journal.pone.0070621 (PMC3734256; doi:10.1371/journal.pone.0070621)
Supplement: Flowchart S1 — Prisma flowchart. (DOC) [file pone.0070621.s001.doc]

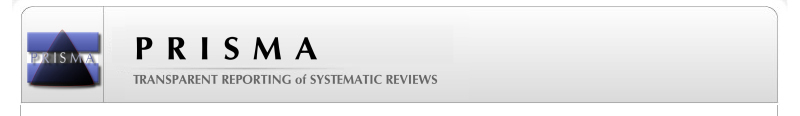
**PRISMA 2009 Flow Diagram**

**Screening**

**Included**

**Eligibility**

**Identification**

Records identified through database searching
(n = 3 )

Additional records identified through other sources
(n = 0 )

Records after duplicates removed
(n =3 )

Records screened
(n = 3 )

Records excluded
(n = 0 )

Full-text articles assessed for eligibility
(n = 3)

Full-text articles excluded, with reasons
(n = 0 )

Studies included in qualitative synthesis
(n = 3)

Studies included in quantitative synthesis (meta-analysis)
(n = 3 )
